# Supplementary material for: Processing Effect and Characterization of Olive Oils from Spanish Wild Olive Trees (Olea europaea var. sylvestris)
Source: Molecules. 2021 Feb 28;26(5):1304. doi: 10.3390/molecules26051304 (PMC7957623; doi:10.3390/molecules26051304)
Supplement: Supplementary file 1 [file molecules-26-01304-s001.pdf]

Analytical standards for the analysis of volatile compounds

39 analytical standards were used: acetic acid, *trans*-2-pentenal, 1-penten-3-one, pentanal, 1-penten-3-ol, pentan-1-ol, 2-methyl-1-butanol, hexanal, *cis*-3-hexen-1-ol, hexan-1-ol, heptanal, octane, octanal, 1-octen-3-ol, *cis*-3-hexenyl acetate, hexyl acetate, *trans*,*trans*-2,4-decadienal, propionic acid, 2-methylpropan-1-ol and butanoic acid, all supplied by *Fluka* (Buchs, Switzerland); 4-methylpentan-2-ol, *trans*,*trans*-2,4-heptadienal, *trans*-2-heptenal, heptan-2-ol, *trans*-2-penten-1-ol, 2-methylbutanal, 3-methylbutanal, 3-methyl butanoic acid, ethyl acetate, *cis*-3-hexenal, 2-methylpropanoic acid, ethyl butanoate, *trans*,*trans*-2,6-nonadienal, and *trans*-2-hexenyl acetate were supplied by *Sigma-Aldrich* (St. Louis, MO, USA); pentan-3-one and nonanal were supplied by *Supelco* (Bellefonte, PN, USA); *cis*-2-penten-1-ol and *trans*-2-hexen-1-ol were supplied by *SAFC* (Darmstadt, Germany); and *trans*-2-hexenal was supplied by *Acrös Organics* (Geel, Belgium).

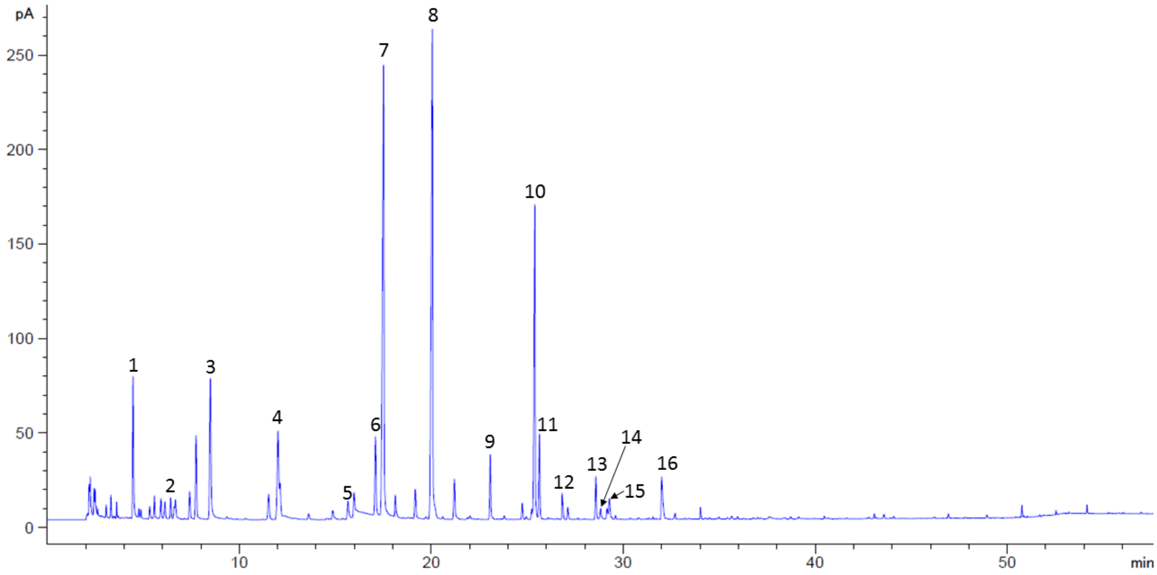

Figure S1. Chromatogram of volatile compounds of sample 1, obtained with the operation conditions of 5.5 mm of sieve hole diameter, 30 °C of malaxation temperature and 60 min of malaxation time. Compounds identified and quantified: 1) ethanol; 2) pentan-3-one; 3) 1-penten-3-one; 4) hexanal; 5) *trans*-2-pentenal; 6) 1-penten-3-ol; 7) 4-methyl-2-pentanol; 8) *trans*-2-hexenal; 9) octanal; 10) *cis*-2-penten-1-ol; 11) *cis*-3-hexenyl acetate; 12) hexan-1-ol; 13) *cis*-3-hexen-1-ol; 14) nonanal; 15) *trans*-2-hexen-1-ol; and 16) acetic acid.
